# Easy Method of Starting Cohesive Gold Fillings

**Published:** 1903-04-15

**Authors:** D. E. Sheehan


### EASY METHOD OF STARTING COHESIVE GOLD FILLINGS.

BY DR. D. E. SHEEHAN.

An easy method of starting cohesive foil fillings is a matter of importance to the dentist and also to the patient; as a time-saver it is most interesting to the former, and as a pain-saver it is appreciated by the latter.

Soft foil may be used as a starter in pits, grooves or in cavities which are merely retentive in form, and because of its plasticity will go to its proper place more readily and more precisely than cohesive foil. In starting a filling a pellet of soft foil is placed in position and firmly driven into the pit or groove. The size of this pellet is governed entirely by conditions. If you start with a pit, only a small pellet is required,if with a groove, a larger pellet. If with the retentive form of the cavity, a large rope of gold made from one-third of a sheet of No. 4 is grasped with the foil carriers and rolled about the pliers between thumb and finger, the whole being pressed into place. Before condensing thoroughly in in any case, a piece of cohesive foil is driven into the center of this first pellet, and the principle is precisely the same as where a metal screw is desired in a stone wall. First, a wooden peg is driven into a hole in the stone, then the screw is driven in the center of the peg, the soft wood undergoing condensation or expansion that it may accommodate the screw, thereby making a powerful resistance which serves to retain it in the wall.

In hypersensitive dentine when excavation is nearly unbearable, the cavity need be made only retentive in form, then by placing a large pellet of soft foil in the cervical region and driving cohesive foil into its center, the desired start is easily made. It is especially indicated in large approximo occlusal cavities in bicuspids and molars, for by filling the cervical third with soft foil, the other two-thirds are easily finished with cohesive. Frequently the cervical third, which is so difficult to perfectly fill, is out of view, and to begin with cohesive foil would be a waste of time and energy, and end in possible failure. So frequently, too, do we find approximal cavities in lower bicuspids which extend into the lingual surface. Here soft foil is to be used in the starting of a filling, cohesive being used to finish if necessary. In occlusal surfaces of molars, where the sulci are burdened with fissures, soft foil to start the cohesive filling will expedite matters, and prevent "balling and rocking" of the gold. In labial and buccal surfaces of the lower teeth, when the operator has often the freedom of but one hand, the indication for soft foil in making the start is very apparent.

Used in the manner described, soft foil as a starter for cohesive foil fillings has always been a pleasure to handle, and resolves itself into a most powerful assistant.

